# Facile fabrication of mesostructured natural rubber/silica nanocomposites with enhanced thermal stability and hydrophobicity

**DOI:** 10.1186/s11671-019-3197-2

**Published:** 2019-12-17

**Authors:** Supphathee Chaowamalee, Chawalit Ngamcharussrivichai

**Affiliations:** 1 Department of Chemical Technology, Faculty of Science, Chulalongkorn University, Pathumwan, Bangkok, 10330 Thailand; 2 Center of Excellence on Petrochemical and Materials Technology (PETROMAT), Chulalongkorn University, Pathumwan, Bangkok, 10330 Thailand; 3 Center of Excellence in Catalysis for Bioenergy and Renewable Chemicals (CBRC), Faculty of Science, Chulalongkorn University, Pathumwan, Bangkok, 10330 Thailand

**Keywords:** Nanocomposite, Mesoporous silica, Natural rubber, *in situ* sol, gel, Surface properties

## Abstract

Natural rubber (NR)/hexagonal mesoporous silica (HMS) nanocomposites (NRHMS) with enhanced thermal and hydrophobic properties were facilely prepared via *in situ* sol–gel formation with pH adjustment using a low sulphuric acid (H_2_SO_4_) acid concentration. The effect of the amount of 0.5 M H_2_SO_4_ (2.5–10 g) added into the pre-synthesis mixture on the physicochemical properties of the obtained NRHMS nanocomposites was investigated. With a small addition of H_2_SO_4_ solution, the fabricated NRHMS nanocomposite possessed an improved wormhole-like mesostructure arrangement with a thicker silica wall, which retarded the thermal decomposition of the NR phase, as deduced from the auto-oxidation of NR by thermogravimetric analysis. The H_2_O adsorption–desorption measurement revealed an increased hydrophobicity of the NRHMS composites, explained by the acid-catalyzed bridging of free silanol groups to siloxane bonds, which was supported by the X-ray photoelectron spectroscopy analysis. Scanning transmission electron microscopy with energy dispersive X-ray spectroscopy elemental mapping revealed a good dispersion of the NR phase within the mesostructured silica. However, a high amount of added H_2_SO_4_ solution led to silica–NR phase separation due to the decreased hydrophobic interaction between the silica precursor and rubber chain, as well as an agglomeration of the NR phase itself. The mechanism of NRHMS nanocomposite formation under pH-controlled conditions was proposed to proceed via a cooperative self-assembly route.

## Introduction

In the past few decades, the soft template-based synthesis method has been utilized to prepare a variety of mesoporous-structured materials [1]. Wormhole-like motif hexagonal mesoporous silica (HMS) has been successfully prepared via a neutral templating based on hydrogen bonding of self-assembled long-chain primary amines and hydrolyzed silica precursors [2]. The choice of HMS reflects its exceptional physicochemical properties compared to conventional periodic mesoporous silica, including an improved thermal and hydrothermal stability due to the thicker silicate wall [3], small domain size with short channels [4, 5], and simplistic template recovery with solvent extraction [6]. In addition, the distinctive sponge-like structure, which exhibits a complementary mesopore framework, improves the framework accessibility and facilitates mass diffusion [7–9]. These advantages have attracted interest in HMS in many promising fields, such as catalysis, drug delivery, and adsorption [10–15].

Modification of HMS surface properties, both chemically and physically, has been widely investigated for preparing unique materials that are suitable for various specific purposes. Hydrophobically modified HMS materials provide a low water affinity on the surface while still possessing its distinctive properties, and so exhibit a good performance in the adsorption of *N*-nitrosamines from tobacco extract solution [16] and controlled release of poorly water-soluble drugs [17]. In addition, an enhanced hydrophobicity improved the hydrothermal stability of the resulting materials [18], making them suitable for applications in aqueous phase solutions. Physical modification of HMS with organic polymers is an interesting method to increase its hydrophobicity due to its relatively easy approach and low cost, and that it provides further possible functionalization of both the silica and organic phases. The preparation of mesoporous silica based-polymer nanocomposites has been broadly summarized into the four methods of (i) blending, a direct mixing of polymer and mesoporous silica by melt or solution blending [19, 20], (ii) *in situ* polymerization, a dispersion of the surface-modified silica phase in monomers followed by polymerization [21, 22], (iii) surface-initiated polymerization, a grafting of polymeric moieties onto mesoporous silica via covalent interaction [23, 24], and (iv) *in situ* sol–gel formation, a direct preparation by either impregnation of polymeric molecules into the silica framework [25] or fabrication of a mesoporous silica/polymer nanocomposite via soft-templated self-assembly [26].

Natural rubber/hexagonal mesoporous silica (NRHMS) nanocomposites synergize the advantages of a mesoporous silica matrix (ordered structure, thermal stability, high surface area, and functionalization simplicity) with the dispersed polyisoprene phase offering hydrophobicity [27]. Moreover, the presence of carbon–carbon double bonds (C=C) in the isoprene structure can potentially be chemically modified via alkene-related reactions, such as electrophilic addition at the C=C [28]. The *in situ* sol–gel synthesis of NRHMS, a one-pot self-assembly synthesis, is a simple strategy under a mild condition and yields a material with desirable properties [27]. The synthesis was performed in the presence of tetrahydrofuran (THF) as the co-solvent, using dodecylamine (DDA) as the structure-directing agent, and tetraethyl orthosilicate (TEOS) as the silica precursor. Despite their high mesoporosity and hydrophobicity, the low thermal stability of the incorporated natural rubber (NR) was a major drawback.

The presence of NR during the mesophase formation hampered the hydrolysis and condensation of the silica precursor, leaving a considerable amount of remnant ethoxy and hydroxyl groups in the resulting nanocomposites, which is detrimental to their physicochemical properties [29, 30]. The rate of hydrolysis and condensation in the formation of silica network strongly relies on the pH [31, 32]. Lin et al. employed a pH-changing method for the synthesis of mesoporous silica nanoparticles [33]. Acidic sulfate and acetate water solution was used for pH adjustment, which resulted in a mesostructured silica with a thicker channel wall and an improved thermal and hydrothermal stability. However, improvement in the thermal stability of NRHMS via pH adjustment has not been studied, yet it is known that addition of a mild acid solution in the sol–gel reaction affects not only the silica network, but also the properties of the NR [34].

In this research, we explored a simple approach for fabricating NRHMS nanocomposites with enhanced thermal stability and hydrophobicity based on pH adjustment using a mild sulfuric acid solution (0.5 M H_2_SO_4_). The effect of the amount of added H_2_SO_4_ on the physicochemical and thermal properties of the resulting NRHMS nanocomposites, as well as the NR phase dispersion, was investigated using various characterization techniques. The chemical environment, in terms of carbon, oxygen, and silica content, provided useful information about the pH-dependent amount of remnant ethoxy and hydroxyl groups. The facile fabrication of NRHMS nanocomposites via this approach gave nanoparticles (NPs) of a homogeneous size, a compact and improved hexagonal mesostructure arrangement, high surface area, and an enhanced hydrophobicity and thermal stability, which are promising characters for catalytic and adsorbent materials.

## Methods

### Materials and chemical reagents

The TEOS and DDA (both AR grade, 98%) were purchased from Sigma Aldrich, while H_2_SO_4_ (98%), THF (99.5%), and absolute ethanol (99.9%) were commercially obtained (AR grade) from QRëC. Ethanol (commercial grade, 95%) was purchased from Alcoh. The NR was supplied by Thai Hua Chumporn Natural Rubber Co. Ltd (Thailand). All chemical reagents and materials were used without further purification.

### Synthesis of pristine HMS

Pristine HMS was synthesized by sol–gel formation as described elsewhere [27]. In a typical batch, 3.75 g of DDA was dissolved in a solution of THF (26.67 g) and deionized water (53.05 g) with stirring and then 10.5 g of TEOS was added dropwise. The mixture was aged with stirring for 1 day at 40 °C. Subsequently, the white solid product was recovered by filtration and dried at 60 °C for 18 h. Template removal was achieved by extraction with 0.05 M H_2_SO_4_/ethanol at 80 °C for 4 h, and the resulting solid was thoroughly washed with ethanol and dried at 60 °C for 12 h.

### Synthesis of NRHMS nanocomposites

A 0.5-g NR sheet was directly swollen in 10.5 g of TEOS overnight. The swollen NR sheet, which took up 2 g of TEOS, was then stirred overnight in 26.67 g THF to obtain a colloidal mixture, to which 3.75 g of DDA and 8.5 g TEOS were sequentially added dropwise with stirring. Next, 53.05 g of deionized water added dropwise and the resulting mixture was stirred and aged at 40 °C for 2 days. The solid product was recovered by precipitation in ethanol, filtration and drying at 60 °C for 18 h. The extraction and product finishing were performed in the same procedure as the HMS synthesis above.

### Synthesis of NRHMS nanocomposites using a low H_2_SO_4_ concentration for pH adjustment

The NR colloidal mixture prepared in the same manner as detailed above for the NRHMS synthesis was aged at 40 °C for 1 day and then the required amount of 0.5 M H_2_SO_4_ (0, 2.55, 5.10, or 10.2 g) was slowly dropped into the mixture under stirring to adjust the pH, and the mixture was further aged for 1 day. The solid product was recovered, extracted, and completed by the same procedure as for NRHMS synthesis. The nanocomposites obtained were designated as NRHMS(*X*), where *X* represents the weight (g) of 0.5 M H_2_SO_4_ added during the synthesis. The weight composition of synthesized materials is summarized in Additional file 1: Table S1.

### Characterization of synthesized materials

The powder X-ray diffraction (XRD) analysis was performed on a Bruker D8 Advanced X-ray diffractometer equipped with Cu Kα radiation operated at an X-ray power of 40 kV and 40 mA. The XRD patterns were recorded at room temperature, scanning from a 2*θ* of 1°–10° at a 0.02° step size and 1 s count time. The characteristic lattice parameter (*a*_0_) was calculated from the interplanar spacing (*d*-spacing) of the (100) reflection peak using the equation: ${a}_0=2{d}_{100}/\sqrt{3}$.

Nitrogen (N_2_) adsorption–desorption measurement at −196 °C was performed on a Mircrometrics ASAP2020 surface area and porosity analyzer to determine the textural properties of the synthesized materials. All samples were degassed at 150 °C for 2 h in the adsorption apparatus before analysis. The specific surface area (*S*_BET_) was calculated using the Brunauer-Emmett-Teller (BET) equation from the adsorption data within the relative pressure (*P/P*_0_) range of 0.02–0.2. External surface area (*S*_ext_) was estimated from the slope of the *t*-plot. The mesopore volume (*V*_m_) was calculated from the intercept of the linear portion of the *t*-plot in the relative pressure range above which N_2_ was condensed inside the primary mesopores. Pore size distribution was determined by the Barrett-Joyner-Halenda (BJH) calculation using the desorption data. The total pore volume (*V*_T_) was attained from the accumulative N_2_ adsorbed volume at *P/P*_0_ of 0.990.

Thermogravimetric analysis (TGA) was used to determine the silica and NR content and the thermal stability of nanocomposites. Each sample (about 10 mg) was heated from 40–850 °C at a ramp rate of 10 °C/min under an air flow (50 mL/min) using a PerkinElmer Pyris Diamond thermogravimetric analyzer.

Fourier-transform infrared spectroscopy (FTIR) was applied to identify the functional groups and NR phase in the synthesized materials. Transmittance FTIR spectra were recorded on a Nicolet iS10 FT-IR spectrometer over the range of 500–4000 cm^-1^ with 64 scans at the resolution of 4 cm^-1^.

The morphology of the samples was examined by field emission scanning electron microscopy (FESEM) using a HITACHI SU-8030 instrument operated at 10 kV on a gold sputtered sample grid. The particle size distribution was measured by ImageJ software. The mesostructured arrangement of materials was observed by transmission electron microscopy (TEM) using a JEOL JEM-2010 transmission electron microscope at an accelerating voltage of 200 kV. The distribution of main elements in the mesoporous material was examined by scanning transmission electron microscopy with energy dispersive X-ray spectroscopy (STEM-EDS) mapping using a JEOL JEM-2010 transmission electron microscope at accelerating voltage of 200 kV under dark field mode.

The chemical state of carbon, silicon, and oxygen on the surface of synthesized materials was analyzed by X-ray photoelectron spectroscopy (XPS) using a Kratos Axis Ultra DLD X-ray photoelectron spectrometer equipped with a monochromic Al Kα X-ray source (1486.7 eV) operated at 15 kV and 5 mA. Survey scans were measured at a spot size of 400 μm and a constant pass energy of 200 eV. The calibration was performed by setting the C1s band at 284.5 eV. The deconvolution of high-resolution XPS element spectra was performed using the XPSPEAK41 software.

## Results and discussion

### NR content and thermal stability

**Fig. 1:**
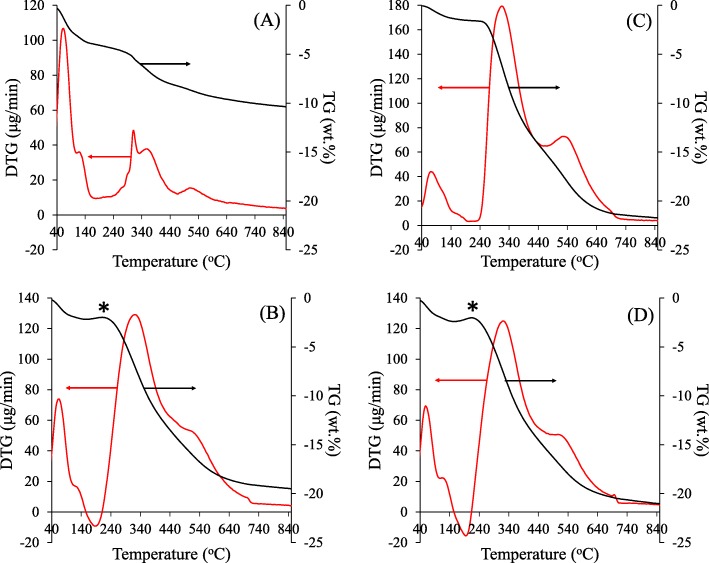
Representative TG and DTG curves of (**a**) HMS, (**b**) NRHMS, (**c**) NRHMS(2.5), and (**d**) NRHMS(10). Asterisk represents the step at which the NR was auto-oxidized

The thermogravimetric (TG) and differential thermogravimetric (DTG) curves of the synthesized materials are shown in Fig. 1. Three major steps of weight loss were observed for all samples. The first step occurred between 40–150 °C, assigned to the loss of ethanol, a by-product from the hydrolysis and condensation of TEOS, and physisorbed water on the material surface. The second weight loss was detected at different temperature ranges for the pristine HMS and NRHMS series. For the pristine HMS (Fig. 1a), the weight loss ranged from 270–450 °C and corresponded to the thermal decomposition of remnant ethoxy groups [35], a typical feature of silica particles prepared by the Stöber method [30]. In case of the NRHMS nanocomposites (Fig. 1), the relatively broad decomposition range extended from 200–450 °C, indicating the decomposition of the NR phase and ethoxy groups. The final weight loss was found in the range of 450–700 °C and corresponded to the dehydroxylation of silanol groups [36] and decomposition of the carbon residue [37]. The amount of NR was determined from the difference in the residue weight between the pristine HMS and NRHMS series. All NRHMS nanocomposites possessed a NR content of 12% by weight (wt.%, Table 1).

**Table 1:** Physicochemical properties of the HMS and NRHMS nanocomposites

Sample	Si content^a^ (wt.%)	NR content^a^ (wt.%)	*S*_BET_^b^ (m^2^ g^-1^)	*S*_ext_^c^ (m^2^ g^-1^)	*V*_T_^d^ (cm^3^ g^-1^)	*V*_P_^e^ (cm^3^ g^-1^)	*D*_p_^f^ (nm)	*d*_100_ (nm)	*a*_o_ (nm)	*W*_T_^g^ (nm)	*V*_m,H2O_^h^ (cm^3^(STP) g^-1^)
HMS	89.8	-	1004	533	2.29	0.47	2.79	4.08	4.71	1.82	70.37
NRHMS	80.4	11.7	648	384	1.37	0.19	2.55	4.20	4.85	2.30	60.15
NRHMS(2.5)	78.2	13.4	577	401	1.50	0.13	2.56	4.66	5.38	2.82	43.32
NRHMS(5)	78.6	13.0	439	315	1.23	0.08	2.55	4.68	5.40	2.85	38.51
NRHMS(10)	78.8	12.9	622	465	1.51	0.10	2.31	4.00	4.61	2.30	43.66

^a^Silica and NR content, determined by TGA

^b^BET surface area

^c^External surface area, determined from the slope of *t*-plot curves

^d^Total pore volume

^e^Mesopore volume, determined from the interception of *t*-plot curves

^f^Pore diameter determined, from BJH method

^g^Wall thickness determined, from the subtraction between pore diameter and unit cell parameter

^h^Monoloyer-adsorbed water, determined from H_2_O adsorption–desorption measurement

It is worth noting that a slight weight gain occurred in the range of 180–200 °C, ascribed to the auto-oxidation of the polymer [38], which occurred in the pristine NRHMS. For NRHMS(2.5) and NRHMS(5), this degradation step was not found, and the initial decomposition temperature of NR was shifted to 240 °C (Fig. 1c and Additional file 1: Figure S1, respectively). It was suggested that the enhanced condensation under the mild acid condition increased the silica framework coverage over the NR, which then limited the accessibility of oxygen to the NR phase and so retarded the decomposition of the entrapped NR chains [39]. However, the auto-oxidation step was observed in NRHMS(10), which possibly implied the limited incorporation of NR into the silicate framework. The NRHMS(10) samples exhibited separated NR agglomerates, as shown in Additional file 1: Figure S2.

### Structural properties analysis

**Fig. 2:**
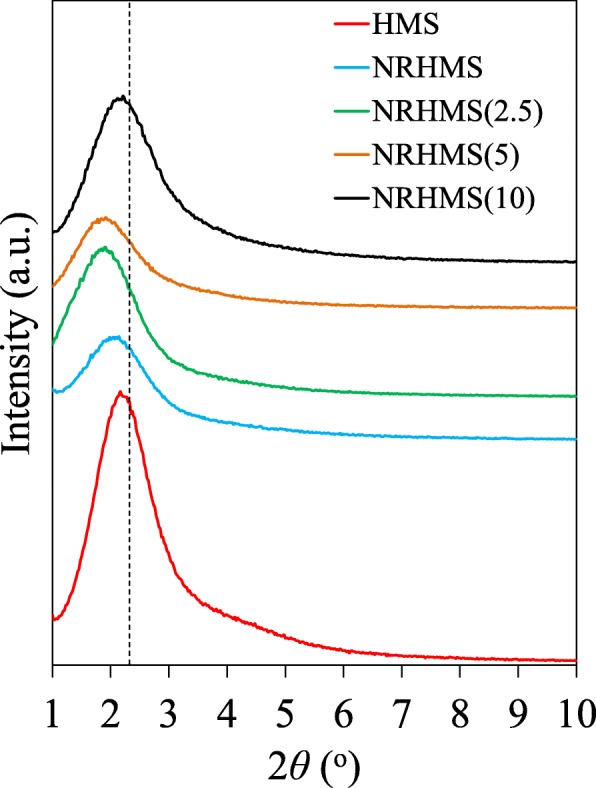
Representative low-angle XRD patterns of HMS and the NRHMS nanocomposites

Low-angle XRD analysis of the HMS and NRHMS series was used to identify the ordered arrangement of the mesostructured silica framework. All the samples possessed the characteristic reflection at a 2*θ* of around 2° (Fig. 2), which was related to *d*_100_ interplanar spacing. Compared to the pure silica HMS, the NRHMS series exhibited a less-ordered mesostructure arrangement, which indicated that the presence of the NR phase in the NRHMS nanocomposites possibly induced the tortuous mesoporous channels. In addition, the characteristic reflection was shifted to lower angles, representing an expansion of the hexagonal unit cell, which indicated the incorporation of NR into the mesostructure silicate framework [27].

The fabrication of NRHMS by adding a low H_2_SO_4_ concentration resulted in an increment in the *d*-spacing and unit cell parameter (*a*_0_) (Table 1). The stronger reflection intensity of NRHMS(2.5), compared to the pristine NRHMS, was ascribed to higher structure ordering due to the acid-catalyzed hydrolysis-condensation rate of silicate species [40]. NRHMS(5) possessed nearly the same unit cell parameter as NRHMS(2.5), but provided a relatively lower ordered mesostructure, suggesting that the dispersion of NR was reduced as the pH of the synthesis mixture decreased [41], conceivably causing the non-uniform incorporation of NR into the hexagonal array silicate framework. For NRHMS(10), the reflection peak was shifted back to a higher position concomitantly with an increased intensity, reflecting that the higher H_2_SO_4_ concentration induced the separation of silicate species and NR phase due to the agglomeration of NR, as mentioned before.

### Textural properties analysis

**Fig. 3:**
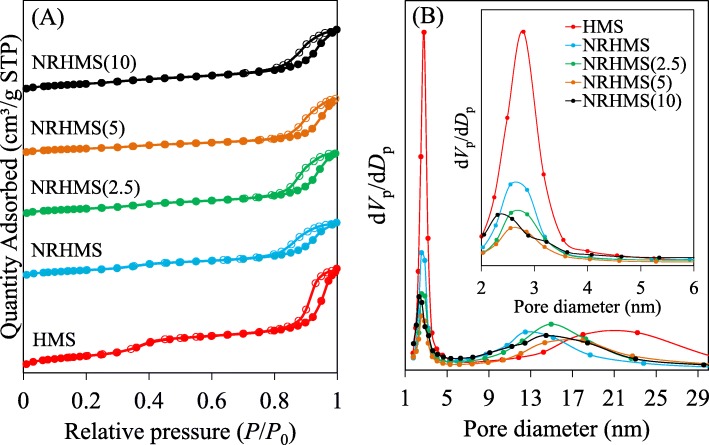
Representative (**a**) N_2_ physisorption isotherms and (**b**) BJH pore size distribution of HMS and the NRHMS nanocomposites

The N_2_ physisorption of the pristine HMS and NRHMS nanocomposites exhibited type IV isotherms (Fig. 3a), indicating the mesoporosity of these materials [42]. There were two main capillary condensation steps at *P*/*P*_0_ ranges of 0.2−0.4 and 0.8−1.0, signifying the presence of a bimodal mesopore distribution, as supported by the BJH plot (Fig. 3b). The smaller pores with a relatively narrow pore size distribution was attributed to the surfactant-templated mesoporous network, while the interparticle voids derived from the NP agglomerates contributed to the larger pores with a broad distribution [43]. Compared to the pristine HMS, the NRHMS series had a lower surface area and primary mesopore volume (Table 1) due to their blockage by NR. The thicker pore wall also caused a decreased surface area and porosity, as described previously [33]. The larger wall thickness of NRHMS affirmed that the rubber chains were entrapped within the silicate framework, while the reduced pore size suggested that some polymeric molecules were positioned within the mesopores.

When compared to the pristine NRHMS, NRHMS(2.5) exhibited a relatively larger wall thickness with a decreased surface area and pore volume, signifying that the addition of a low H_2_SO_4_ concentration promoted the condensation of silicate species, thickening the silica wall. NRHMS(5) had a slightly thickened pore wall, but still a noticeably decreased surface area and pore volume, which was related to the partial agglomeration of NR, as described in the XRD results. For NRHMS(10), the phase separation caused significant changes in the textural properties, providing a high surface area, but lower unit cell and pore wall thickness as less NR phase was incorporated within the silicate framework. In addition, its pore size was smaller than in the other NRHMS samples because the addition of the low H_2_SO_4_ concentration led to an increased polarity of the synthesis mixture, which decreased the size of micelles due to a weakened interaction between the micelles themselves [44].

### Morphology and porous structure analysis

**Fig. 4:**
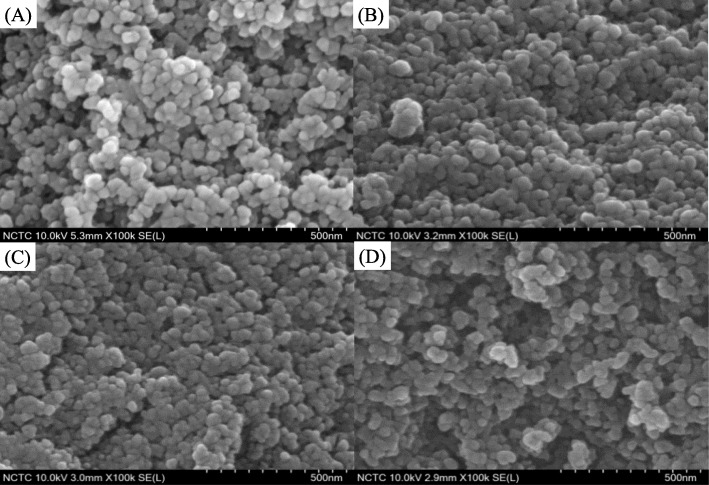
Representative FESEM images of (**a**) HMS, (**b**) NRHMS, (**c**) NRHMS(2.5), and (**d**) NRHMS(10) at a magnification of ×100,000

The FESEM analysis revealed that the siliceous HMS formed spherical silica NP aggregates, providing noticeably interparticle voids (Fig. 4). For the NRHMS series, the presence of NR possibly acted as binding modules, attaching particles into a closer packed arrangement. As a result, the NRHMS samples had smaller interparticle voids than HMS, which supported the BJH pore size distribution (Fig. 3b). NRHMS(2.5) possessed a similar morphology to the pristine NRHMS, while the morphology of NRHMS(10) looked similar to that of HMS, supporting the low level of NR incorporation into the silicate framework due to NR aggregation. From the particle size distribution measurement (Additional file 1: Figure S3), NRHMS and NRHMS(2.5) exhibited larger size than HMS. The addition of acid solution led to somewhat enlarged particle sizes by an increased rate in the silicate framework formation, except for NRHMS(10) that provided smaller particle size than the pristine NRHMS due to the less amount of incorporated and bound NR.

**Fig. 5:**
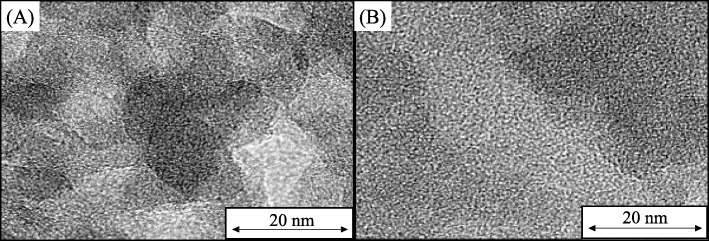
Representative TEM images of HMS (**a**) and NRHMS (**b**) at a magnification of ×300,000

The TEM analysis of HMS and representative NRHMS samples (Fig. 5) revealed the pure silica HMS and the NRHMS series possessed a wormhole-like motif structure, a prominent feature of HMS [5]. This indicated the retention of the mesostructured silicate framework in the HMS matrix with entrapped NR molecules. Unfortunately, the conventional TEM mode could not detect the dispersed NR phase because of the low atomic weight of the constituted elements. Besides, the mesopores of synthesized HMS and NRHMS materials were difficult to be measured due to their tortuous mesostructured framework and the absence of long-range mesopore ordering. It was suggested that HMS exhibited wormhole-like structure with local hexagonal symmetry [45].

**Fig. 6:**
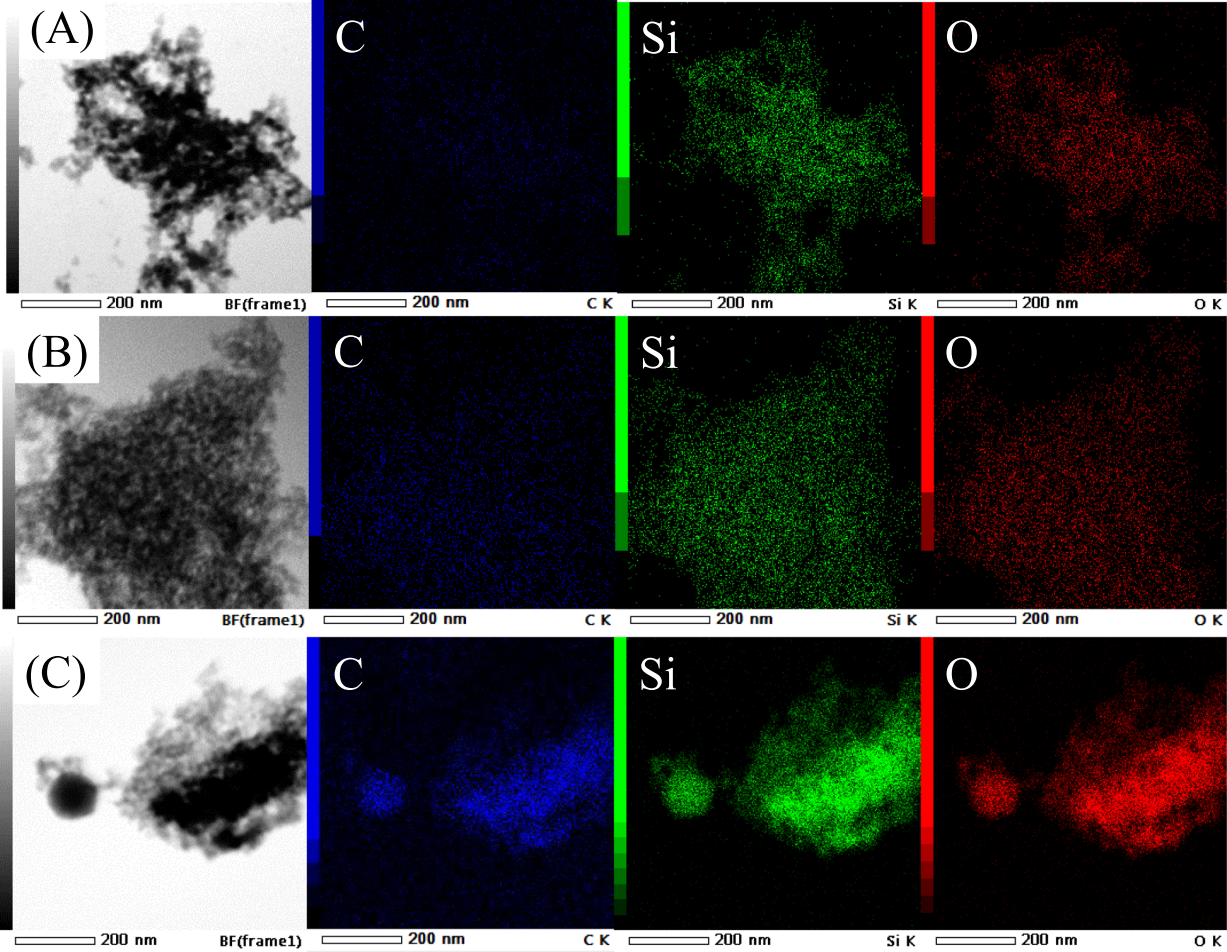
Representative STEM with EDS mapping images of (**a**) HMS, (**b**) NRHMS, and (**c**) NRHMS(10) at a magnification of ×200,000

The fabricated nanocomposites were, therefore, further characterized by STEM-EDS to locate the whereabouts of the NR phase (Fig. 6). The pristine HMS had a uniform distribution of silicon (Si) and oxygen (O), corresponding to the silicate framework [23], while a small amount of carbon (C) was detected, which was explained by the presence of remnant ethoxy groups. The pristine NRHMS provided a higher amount of C, which was consistently detected in the NRHMS nanocomposites, indicating that the NR phase was uniformly dispersed in its mesostructure. In contrast, a large cluster of C was detected for the NRHMS(10) with separated NR agglomerates.

**Fig. 7:**
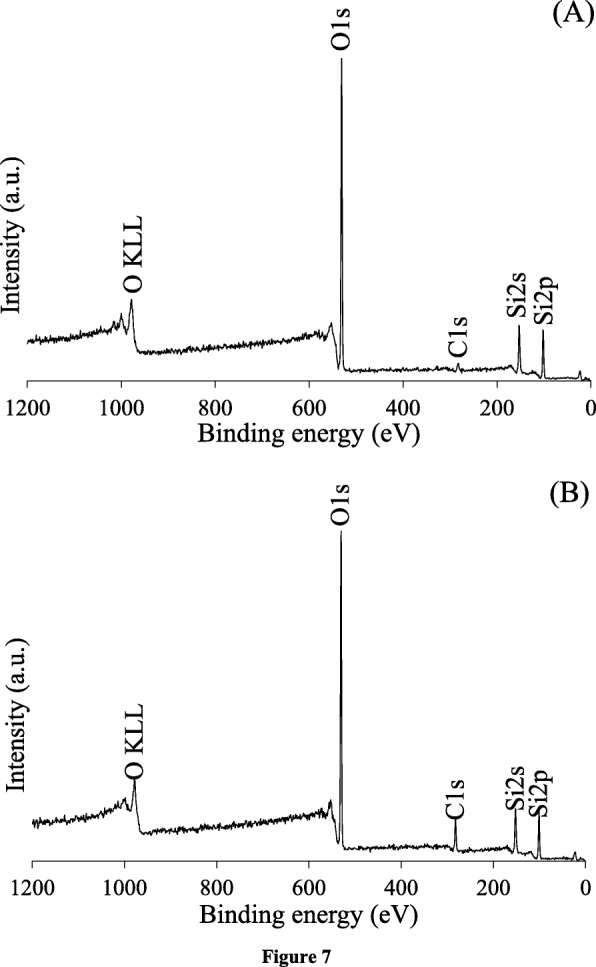
Representative wide scan XPS spectra of (**a**) HMS and (**b**) NRHMS

The chemical environment on the surface of materials was evaluated by XPS analysis. The wide scan XPS spectrum of the siliceous HMS (Fig. 7a) revealed four distinctive bands at a binding energy (BE) of 284.5, 533.0, 151.0, and 100.0 eV, which were assigned to C1s, O1s, Si2s, and Si2p, respectively. From Fig. 7b, elemental composition of NRHMS provided a similar characteristic pattern to the HMS, supporting that there was no other elemental contamination in the presence of NR.

**Fig. 8:**
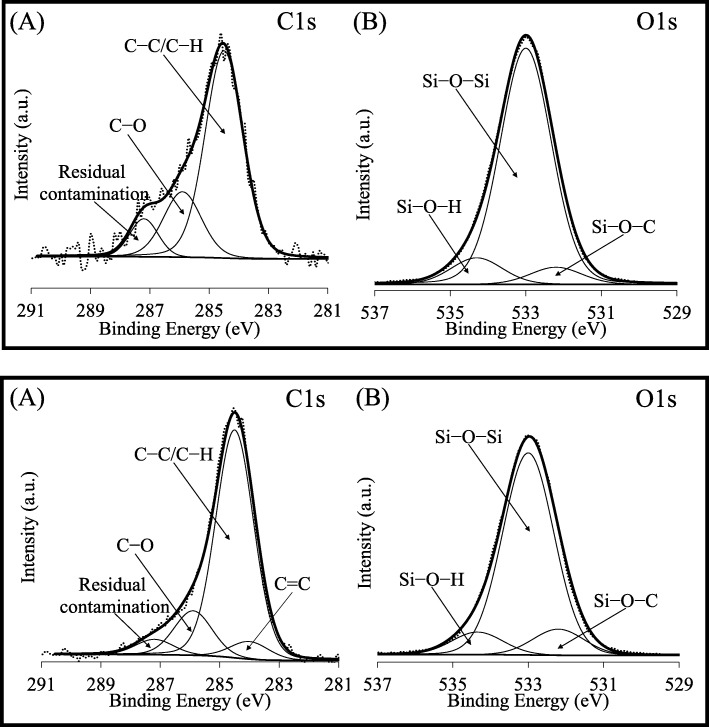
Representative core level high-resolution C1s and O1s spectra of (**a**) HMS and (**b**) NRHMS

The high-resolution C1s and O1s spectra of HMS are shown in Fig. 8a. The first chemical state at a BE of 284.5 eV corresponded to the C–C/C–H bonds of aliphatic hydrocarbons in the sample and adventitious surface carbon. The band at a BE of 285.9 eV was ascribed to the C–O species of remnant ethoxy groups, while a small deconvoluted band at a BE of 287.2 eV was related to residual contamination during synthesis [46]. For the high-resolution O1s spectrum, the Si–O–Si bonds of the silicate network contributed the BE of 533.0 eV. Two additional components were detected at a BE of 532.2 and 534.3 eV, corresponding to the Si–O–C bonds of remnant ethoxy groups and the Si–O–H of silanol groups, respectively.

The pristine NRHMS exhibited a similar high-resolution C1s spectrum to HMS, but with the C=C species of polymeric rubber structure at a BE of 284.0 eV (Fig. 8b). According to the obtained O1s spectra, NRHMS possessed the highest relative concentration of Si–O–C, when compared to HMS, implying that the presence of NR indeed hampered the hydrolysis of the silica precursor by reason of the interaction between ethoxy groups and rubber molecules [47]. From Additional file 1: Figure S5, the C1s and O1s states of NRHMS(2.5) and NRHMS(5) were similar to those of the pristine NRHMS. This result supported that the addition of a low H_2_SO_4_ concentration enhanced the thermal stability of the fabricated NRHMS nanocomposite by entrapping NR between the thickened silica wall, not by chemical bonding between NR molecules and the silicate framework.

Table 2 summarizes the atomic percentage (at%) for the C1s, O1s, and Si2p core peaks observed in the HMS and NRHMS nanocomposites. When the H_2_SO_4_ was added into the NR/HMS mixture during synthesis, the amount of remnant ethoxy groups and silanol groups decreased while the siloxane species increased. The result supported that addition of a low H_2_SO_4_ concentration enhanced the hydrolysis and condensation rate of silicate species. In addition, the decreased amount of silanol groups possibly resulted from sulfate ions (SO_4_^2-^) effectively binding to the hydroxyl groups on the surface to form siloxane bonds [48].

**Table 2:** XPS binding energies and atomic percentage for the C1s, O1s, and Si2p core peaks of HMS and the NRHMS nanocomposites

Core peak	HMS			NRHMS			NRHMS(2.5)			NRHMS(5)		
	BE/eV (FWHM/eV)	at%		BE/eV (FWHM/eV)	at%		BE/eV (FWHM/eV)	at%		BE/eV (FWHM/eV)	at%	
C1s												
C=C	-	-		284.0 (1.5)	0.50		284.0 (1.5)	0.42		284.0 (1.5)	0.53	
C–C	284.5 (1.5)	2.00		284.5 (1.5)	6.42		284.5 (1.5)	6.12		284.5 (1.5)	6.72	
C–O	285.9 (1.5)	0.63		285.9 (1.5)	1.25		285.9 (1.5)	1.16		285.9 (1.5)	0.81	
Carbon residue	287.2 (1.5)	0.26	2.89	287.2 (1.5)	0.43	8.11	287.2 (1.5)	0.37	8.07	287.2 (1.5)	0.24	
Total											8.30	
O1s												
Si–O–C	532.2 (1.6)	5.10		532.2 (1.6)	8.07		532.2 (1.6)	7.25		532.2 (1.6)	6.01	
Si–O–Si	533.0 (1.6)	70.35		533.0 (1.6)	62.52		533.0 (1.6)	65.56		533.0 (1.6)	68.50	
Si–O–H	534.3 (1.6)	7.89	83.34	534.3 (1.6)	6.98	77.57	534.3 (1.6)	6.24	79.06	534.3 (1.6)	5.06	79.57
Total												
Si2p												
Si2 p_3/2_	103.5 (1.7)	9.18		103.5 (1.7)	9.55		103.5 (1.7)	8.58		103.5 (1.7)	8.09	
Si2 p_1/2_	104.1 (1.7)	4.59	13.77	104.1 (1.7)	4.78	14.33	104.1 (1.7)	4.29	12.87	104.1 (1.7)	4.04	12.13
Total												

### Hydrophobicity measurement

Given that NR is a hydrophobic polymer, its incorporation into the mesostructure silicate framework provided the resulting NRHMS nanocomposite with hydrophobic properties. Table 1 shows the monolayer-adsorbed volume of water (*V*_m,H2O_), as obtained from the H_2_O adsorption–desorption measurement. The pristine HMS possessed the highest affinity for water because of the presence of silanol groups as the main functional groups on its surface. The hydrophobicity of the incorporated NR phase reduced the water affinity of the NRHMS nanocomposites. For NRHMS(2.5) and NRHMS(5), an increased hydrophobicity was observed since the addition of the low H_2_SO_4_ concentration reduced the amount of silanol groups via acid-catalyzed condensation to siloxane bonds, which considerably decreased the water affinity (Table 2) [49]. However, the unsuccessful incorporation of NR, which occurred in NRHMS(10), resulted in a decreased hydrophobicity since the hydrophilicity from the silicate framework was more dominant than the NR agglomerates.

### Mechanism of NRHMS nanocomposite formation

**Scheme 1:**
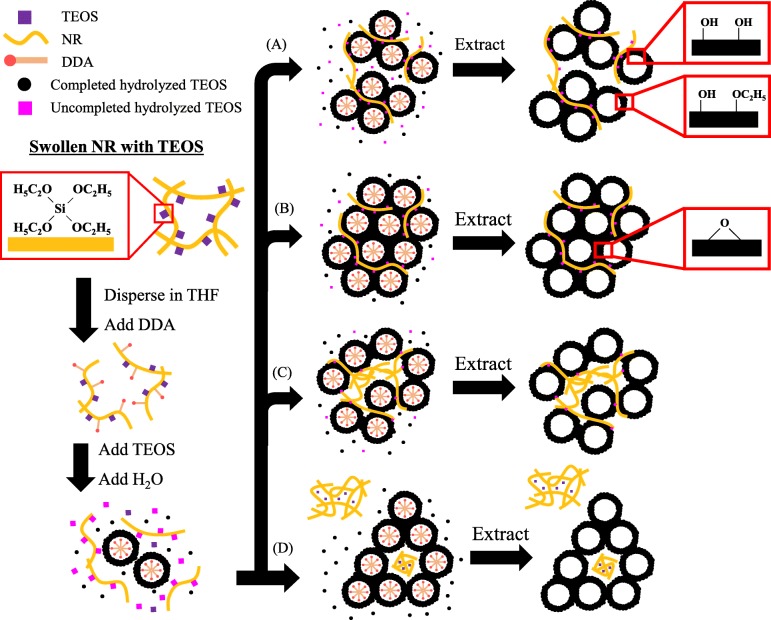
Mechanistic aspect for the formation of (**a**) pristine NRHMS, (**b**) NRHMS(2.5), (**c**) NRHMS(5) and (**d**) NRHMS(10)

The mechanistic formation of the NRHMS nanocomposites has been reported previously [47], where NR was incorporated within the silica framework due to hydrophobic interactions between the poly(cis 1,4-isoprene) molecules and the ethoxy groups of the partially hydrolyzed silica precursor, forming a hybrid mesophase via a cooperative self-assembly route. The NRHMS nanocomposite exhibited inferior physical properties compared to the siliceous HMS, but a superior hydrophobicity due to the NR being uniformly dispersed within the silicate framework. However, the presence of remnant ethoxy groups suggested that the rubber phase hindered the hydrolysis and condensation of the silica precursor (TEOS), resulting in a less condensed and ordered mesostructured silicate network (Scheme 1A). After fabrication in the presence of low H_2_SO_4_ concentration (Scheme 1B), the amount of ethoxy groups and silanol groups was decreased via the acid-catalyzed sol–gel process, resulting in an improved ordered mesostructure with thicker pore walls entrapping the rubber chain. The coverage of NR on the silica wall prevented the decomposition of polymeric molecules via auto-oxidation, as seen in the TGA result (Fig. 1). In addition, the decreased amount of silanol groups on the surface improved the hydrophobicity of the resulting fabricated NRHMS. Scheme 1C illustrates that the higher H_2_SO_4_ concentration in the synthesis mixture decreased the dispersion stability of NR, since the pH reduction destabilized the negative charges around the small NR particles, allowing them to agglomerate [50]. Simultaneously, the decreased pH (increased the amount of acid solution added) rapidly promoted the condensation of the silica precursor, potentially causing silicate species to form a mesostructure with less incorporated NR. This catalytic effect also diminished the level of ethoxy groups, which acted as anchoring points between the silicate species and NR molecules. As a result, the previous entrapped NR molecules, which were directly exposed to the mixture environment, partially coiled within the mesostructured silica framework, and hampered the formation of an ordered hexagonal mesophase. At higher amount of acid solution added, separation of the NR phase and silicate framework became more obvious (Scheme 1D).

## Conclusion

We successfully demonstrated a facile fabrication of NRHMS nanocomposites via the *in situ* sol–gel method with pH adjustment to different acid levels using 0.5 M H_2_SO_4_ solution. Compared to the pristine NRHMS, NRHMS(2.5) exhibited an increased pore wall thickness and hexagonal mesostructure ordering, since the addition of H_2_SO_4_ at a low concentration, enhanced the acid-catalyzed sol–gel reaction of silicate species, while retaining the remnant ethoxy groups derived from TEOS as anchoring points between the silicate and NR phase. The strengthened silica wall also entrapped the NR chains within its framework, which retarded the thermal decomposition of NR. The improved hydrophobicity of the fabricated nanocomposite was explained by the acid-induced condensation of surface silanol groups to siloxane bonds. However, the advantages of adding H_2_SO_4_ at a low concentration were reduced with increasing the amount of acid solution added by the agglomeration of the NR phase and depletion of remnant ethoxy groups, leading to less entrapped NR chains within the mesostructured silicate framework and eventually to phase separation between the NR and silica phases, as observed for the NRHMS(5) and NRHMS(10) nanocomposites, respectively. The fabricated NRHMS nanocomposites with enhanced thermal and hydrophobic properties would be a potential porous carrier in the field of catalysis, adsorption, and drug delivery.

## Supplementary information

**Additional file 1:** Characteristics of NR/HMS nanocomposites.

## Data Availability

The datasets analysed during the current study are available from the corresponding author on reasonable request.

## Abbreviations

DDA: Dodecylamine
DTG: Differential thermogravimetric
FESEM: Field emission scanning electron microscopy
FTIR: Fourier-transform infrared spectroscopy
HMS: Hexagonal mesoporous silica
NP: Nanoparticle
NR: Natural rubber
NRHMS: Natural rubber/hexagonal mesoporous silica nanocomposites
STEM-EDS: Scanning transmission electron microscopy with energy dispersive X-ray spectroscopy
TEM: Transmission electron microscopy
TEOS: Tetraethyl orthosilicate
TG: Thermogravimetric
TGA: Thermogravimetric analysis
THF: Tetrahydrofuran
XPS: X-ray photoelectron spectroscopy
XRD: X-ray diffraction
