# Supplementary material for: Facile fabrication of mesostructured natural rubber/silica nanocomposites with enhanced thermal stability and hydrophobicity
Source: Nanoscale Res Lett. 2019 Dec 17;14:382. doi: 10.1186/s11671-019-3197-2 (PMC6917676; doi:10.1186/s11671-019-3197-2)
Supplement: Supplementary file 1 — Additional file 1: Characteristics of NR/HMS nanocomposites. [file 11671_2019_3197_MOESM1_ESM.docx]

**Supplementary Materials**

**Facile fabrication of mesostructured natural rubber/silica nanocomposites with enhanced thermal stability and hydrophobicity**

**Supphathee Chaowamalee^a,b^ and Chawalit Ngamcharussrivichai ^a,b,c,*^**

^a^ *Department of Chemical Technology, Faculty of Science, Chulalongkorn University,*

*Pathumwan, Bangkok 10330, Thailand*

^b^ *Center of Excellence on Petrochemical and Materials Technology (PETROMAT),*

*Chulalongkorn University, Pathumwan, Bangkok 10330, Thailand*

^c^ *Center of Excellence in Catalysis for Bioenergy and Renewable Chemicals (CBRC),*

*Faculty of Science, Chulalongkorn University, Pathumwan, Bangkok 10330, Thailand*

**Corresponding author: Tel.: +66-2-218-7528; Fax: +66-2-255-5831;*

*E-mail: Chawalit.Ng@Chula.ac.th*

**Table S1** Weight composition of respective synthesis condition

| Sample | Weight composition (g) | | | | | | Final pH of mixture |
| --- | --- | --- | --- | --- | --- | --- | --- |
|  | NR | THF | DDA | TEOS | H_2_O | 0.5 M H_2_SO_4_ |  |
| HMS | - | 26.67 | 3.75 | 10.5 | 53.05 | - | 8.9 |
| NRHMS | 0.5 | 26.67 | 3.75 | 10.5 | 53.05 | - | 9.1 |
| NRHMS(2.5) | 0.5 | 26.67 | 3.75 | 10.5 | 53.05 | 2.55 | 8.1 |
| NRHMS(5) | 0.5 | 26.67 | 3.75 | 10.5 | 53.05 | 5.10 | 7.7 |
| NRHMS(10) | 0.5 | 26.67 | 3.75 | 10.5 | 53.05 | 10.20 | 6.9 |

**Figure S1** Representative TG and DTG curves of NRHMS(5).

**Figure S2** Representative image of the NRHMS(10) nanocomposite with unincorporated NR

(in red circle).

**Figure S3** Representative FESEM images at a magnification of 100000× and

particle size distribution of (A) HMS, (B) NRHMS, (C) NRHMS(2.5) and (D) NRHMS(10).

**Figure S4** Representative wide scan XPS spectra of (A) NRHMS(2.5) and (B) NRHMS(5).

**Figure S5** Representative core level high resolution C1s and O1s spectra of (A) NRHMS(2.5) and (B) NRHMS(5).

**Figure S6** Representative FTIR spectra of the HMS and NRHMS nanocomposites.

From Figure S6, the main strong band was observed between 1000 and 1300 cm^-1^, representing the asymmetric stretching of siloxane bond (Si−O−Si). The small band at 780 cm^-1^ was assigned to the symmetric Si−O stretching of the silica framework, while the band at 940 cm^-1^ was ascribed to the stretching vibration of Si−OH bond. HMS exhibited a relatively small band of C−H stretching, confirming the presence of non-hydrolyzed ethoxy groups in the silica framework. Another strong band located around 3500 cm^-1^ was assigned to hydrogen bonded silanol groups. For the NRHMS series, the C−H stretching band was detected at a relatively high intensity compared to the pristine HMS between 2800 cm^-1^ and 3000 cm^-1^. The characteristic bands of NR at 1370 cm^-1^ and 1440 cm^-1^ were also observed, indicating the presence of NR in the nanocomposites.
